# Developmental dysfunction of prefrontal–hippocampal networks in mouse models of mental illness

**DOI:** 10.1111/ejn.14436

**Published:** 2019-06-11

**Authors:** Victoria C. Oberlander, Xiaxia Xu, Mattia Chini, Ileana L. Hanganu‐Opatz

**Affiliations:** ^1^ Developmental Neurophysiology Institute of Neuroanatomy University Medical Center Hamburg‐Eppendorf Hamburg Germany; ^2^ Present address: BABA Center Pediatric Research Center Helsinki University Hospital Helsinki Finland Helsinki Finland

**Keywords:** development, oscillations, prefrontal–hippocampal network, schizophrenia, synchrony

## Abstract

Despite inherent difficulties to translate human cognitive phenotype into animals, a large number of animal models for psychiatric disorders, such as schizophrenia, have been developed over the last decades. To which extent they reproduce common patterns of dysfunction related to mental illness and abnormal processes of maturation is still largely unknown. While the devastating symptoms of disease are firstly detectable in adulthood, they are considered to reflect profound miswiring of brain circuitry as result of abnormal development. To reveal whether different disease models share common dysfunction early in life, we investigate the prefrontal–hippocampal communication at neonatal age in (a) mice mimicking the abnormal genetic background (22q11.2 microdeletion, DISC1 knockdown), (b) mice mimicking the challenge by environmental stressors (maternal immune activation during pregnancy), (c) mice mimicking the combination of both aetiologies (dual‐hit models) and pharmacological mouse models. Simultaneous extracellular recordings in vivo from all layers of prelimbic subdivision (PL) of prefrontal cortex (PFC) and CA1 area of intermediate/ventral hippocampus (i/vHP) show that network oscillations have a more fragmented structure and decreased power mainly in neonatal mice that mimic both genetic and environmental aetiology of disease. These mice also show layer‐specific firing deficits in PL. Similar early network dysfunction was present in mice with 22q11.2 microdeletion. The abnormal activity patterns are accompanied by weaker synchrony and directed interactions within prefrontal–hippocampal networks. Thus, only severe genetic defects or combined genetic environmental stressors are disruptive enough for reproducing the early network miswiring in mental disorders.

## Graphical abstract

Patterns of network and firing activity as well as the directed interactions between prefrontal cortex and hippocampus are impaired at neonatal age in one‐hit Df16 and dual‐hit genetic environmental, but not one‐hit DISC1, one‐hit environmental (MIA) and pharmacological (KET) mouse models of mental illness.

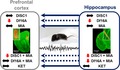

**Abbreviations**
Df(16)A^+/−^: bioengineered knockout mice mimicking the 22q11.2 microdeletion
Df16: Df(16)A^+/−^ model
DISC1: Disrupted‐In‐Schizophrenia‐1
dual‐hit: mice mimicking two risk factors
G: gestational day
GPDC: generalized partial directed coherence
HP: hippocampus
i/vHP: intermediate/ventral hippocampus
KET: ketamine model
LFP: local field potential
MIA: maternal immune activation
MUA: multiple unit activity
one‐hit: mice mimicking one risk factor
PFC: prefrontal cortex
PL: prelimbic cortex
poly I:C: polyriboinosinic polyribocytidilic acid
P: post‐natal day

## 1 INTRODUCTION

Modelling of human neuropsychiatric disorders in animals has been proven to be extremely challenging due to (a) uniqueness of human symptoms, such as hallucinations, guilt, delusions, that cannot be identified in animals, (b) the arbitrary boundaries between normal behaviour and distinct disorders and the lack of objectively ascertainable manifestations and (c) the poor mechanistic insights at molecular, cellular and system level (Kaiser, Zhou, & Feng, 2017; Nestler & Hyman, 2010). Despite these inherent difficulties, a wealth of models has been investigated for shedding light on some aspects of major neuropsychiatric disorders, such as schizophrenia, bipolar disorder and depression. These models need to provide construct, face and predictive validity. Construct validity refers to the ability of the model to mimic the aetiology of disease. Numerous genetic linkages have been established for schizophrenia, yet many of them exert only small effects on human disease risk. For example, deficient Disrupted‐in‐Schizophrenia‐1 (DISC1) has been initially highlighted a risk factor of disease but it rather orchestrates molecular cascades hypothesized to underlie disease‐relevant physiological and behavioural abnormalities (Cuthbert & Insel, 2013). However, unequivocal risk alleles, such as the microdeletion on human chromosome 22 (22q11.2) have also been identified and hereupon mimicked in the Df(16)A^+/−^ mouse line (International Schizophrenia, 2008). Construct validity might be also achieved through exposure of mice to well‐validated environmental risk factors. Among others, maternal immune activation (MIA) has been identified as primer for a large spectrum of neuropsychiatric disorders (Estes & McAllister, 2016). The effects of environmental risk factors, mainly acting during development, depend on the genetic background (Owen, Sawa, & Mortensen, 2016). Gene–environment interactions augment the disease risk (Nimgaonkar, Prasad, Chowdari, Severance, & Yolken, 2017; van Os et al., 2014). Face validity refers to the ability of an animal model to recapitulate the feature of human disorder. For example, Df(16)A^+/−^ mice show abnormal long‐range coupling in the brain during working memory tasks similarly to schizophrenia patients (Meyer‐Lindenberg et al., 2005; Schwarz, Tost, & Meyer‐Lindenberg, 2016; Sigurdsson, Stark, Karayiorgou, Gogos, & Gordon, 2010). Predictive validity refers to the ability of mouse models to respond to pharmacological treatments similarly to patients. However, in the absence of mechanistic understanding of most psychiatric disorders, it is still difficult to develop reliable models of drug action.

The large variety and number of mouse models of neuropsychiatric disorders lead to the question whether they share common mechanisms of network dysfunction for a specific behavioural defect (Hamm, Peterka, Gogos, & Yuste, 2017). Impairment of memory and executive abilities is a core feature of these disorders and, in contrast to positive symptoms (e.g., hallucinations, delusions), detectable in animal models. It relies on abnormal communication within a large network centred on PFC and i/vHP (Sigurdsson & Duvarci, 2015). This network dysfunction seems to emerge early in life, long before the first clinical symptoms at juvenile‐young adult age. We recently showed that the initial coupling between PL and i/vHP of heterozygous DISC1 mice experiencing MIA is impaired shortly after birth, a weaker hippocampal drive being not able to entrain locally miswired prefrontal circuits (Hartung et al., 2016; Xu, Chini, Bitzenhofer, & Hanganu‐Opatz, 2019). Correspondingly, the mice show poorer recognition memory at juvenile age. The prefrontal–hippocampal coupling emerging during neonatal development might be similarly impaired in other disease models that show equally impaired cognitive behaviour. To test this hypothesis, we investigated the patterns of electrical activity, the synchrony and directed interactions between PFC and HP of one‐hit genetic (DISC1^+/−^, Df(16)A^+/−^), one‐hit environmental (mimicking MIA), dual‐hit (combined DISC1^+/−^ or Df(16)A^+/−^ and MIA) and pharmacological model (treatment with ketamine) mice at neonatal age (post‐natal day (P) 8–10). We show that early prefrontal–hippocampal dysfunction is mainly present in one‐hit Df(16)A^+/−^ and dual‐hit models.

## 2 MATERIALS AND METHODS

### 2.1 Animal models

Experiments were performed in compliance with the German laws and the guidelines of the European Community for the use of animals in research and were approved by the local ethical committee Behörde für Gesundheit und Verbraucherschutz of City Hamburg (proposal number 132/12 and 015/18). Timed pregnant mice were obtained at gestational day (G) 6–7 from the animal facility of the University Medical Center Hamburg‐Eppendorf and housed individually at a 12 hr light/12 hr dark cycle, with access to water and food ad libitum. The day of vaginal plug detection was considered as G0.5, while the day of birth as P0. Heterozygous genetically engineered mutant DISC1 mice carrying a Disc1 allele (Disc1^Tm1Kara^) on a C57Bl6/J background were used as one‐hit genetic model (DISC1, *n* = 17; *n* = 5 P8 pups, *n* = 6 P9 pups, *n* = 6 P10 pups). Due to two termination codons and a premature polyadenylation site, the allele produces a truncated transcript (Kvajo et al., 2008). Genotypes were determined using genomic DNA and following primer sequences: forward primer 5′‐TAGCCACTCTCATTGTCAGC‐3′, reverse primer 5′‐CCTCATCCCTTCCACTCAGC‐3′. As a second one‐hit genetic model the Df(16)A^+/−^ model was used (Stark et al., 2008). The affected allele in the Df(16)A^+/–^ mice (Df16, *n* = 11; *n* = 3 P8, *n* = 5 P9, *n* = 3 P10) carries a chromosomal engineered 1.3‐Mb microdeletion ranging from Dgcr2 to Hira, a segment syntenic to the 1.5‐Mb human 22q11.2 microdeletion that encompasses 27 genes. Genotypes were determined using genomic DNA and following primer sequence indicating a knockout: forward primer 5′‐ATTCCCCATGGACTAATTATGGACAGG‐3′, reverse primer 5′‐GGTATCTCCATAAGACAGAATGCTATGC‐3′. The offspring of pregnant dams injected i.v. at G9 with the viral mimetic polyinosinic:polycytidylic acid (poly I:C, 5 mg/kg) were used as one‐hit environmental model (MIA, *n* = 10; *n* = 3 P8, *n* = 4 P9, *n* = 3 P10), since they showed at adulthood deficits highly reminiscent of schizophrenia (Meyer & Feldon, 2012; Meyer, Feldon, Schedlowski, & Yee, 2006). The heterozygous offspring of DISC1^+/−^ or of Df(16)A^+/−^ dams injected at G9 with poly I:C were used as dual‐hit genetic environmental models (DISC1 + MIA, *n* = 17; *n* = 4 P8, *n* = 6 P9, *n* = 7 P10; Df16 + MIA, *n* = 11; *n* = 3 P8, *n* = 6 P9, *n* = 2 P10). Pups chronically treated with ketamine (60 μg/g body weight/day) from P1 to P8 were used as pharmacological model (KET, *n* = 16; *n* = 5 P8, *n* = 7 P9, *n* = 4 P10; Behrens et al., 2007). Non‐treated wild‐type mice (control, *n* = 23; *n* = 11 P8, *n* = 8 P9, *n* = 4 P10) and mice injected with saline (0.9%; saline, *n* = 13; *n* = 4 P8, *n* = 9 P9, *n* = 5 P10) were used as controls. All mice used in this study were generated on C57Bl6/J background (Jackson Laboratories, Bar Harbor, Maine, USA). Pups were investigated during neonatal development at P8–10, the time period of maximal unidirectional hippocampal–prelimbic interactions (Brockmann, Poschel, Cichon, & Hanganu‐Opatz, 2011). During neonatal development, the weight of pups was similar for all eight groups (control: 5 ± IQR 1.1 g; saline: 4.9 ± IQR 0.5 g; DISC1: 4.8 ± IQR 1.3; Df16: 4.8 ± IQR 1.0 g; MIA: 4.1 ± IQR 0.8 DISC1 + MIA: 4.3 ± IQR 1.0 g; Df16 + MIA: 4.5 ± IQR 0.8 g, KET: 4.7 ± IQR 0.6 g, *H* ^2^ = 0.075 *p *=* *0.78 [Kruskal–Wallis]). All investigated groups were age (*H* ^2^ = 4.56, *p *=* *0.713, Kruskal–Wallis) and sex (χ^2^ = 3.38, *p *=* *0.848, chi‐square) balanced.

### 2.2 Electrophysiological recordings in vivo

Multi‐site extracellular recordings were performed in the PL and i/vHP of P8–10 pups of both sexes. Mice were injected i.p. with urethane (1 mg/g body weight; Sigma‐Aldrich) before surgery. Under isoflurane anaesthesia (induction: 5%; maintenance: 2.5%), the head of the pup was fixed into a stereotaxic apparatus using two plastic bars mounted on the nasal and occipital bones with dental cement. The bone over the PFC (0.8 mm anterior to bregma, 0.1–0.5 mm right to the midline) and the i/vHP (3.5–3.7 mm anterior to bregma, 3.5–3.8 mm right to the midline) was carefully removed by drilling holes of <0.5 mm in diameter. Four‐shank electrodes (4 × 4 recording sites, 0.3–2.2 MΩ impedance, 100 μm spacing, 125 μm inter‐shank spacing, NeuroNexus) were inserted into PL at a depth of 1.9 mm from the skull surface. One‐shank electrodes (1 × 16 recording sites, 0.3–2.2 MΩ impedance, 50 μm spacing, NeuroNexus) were inserted into the i/vHP until a depth of 1.3–1.8 mm from the skull surface, at an angle of 20° from the vertical plane. Electrodes were labelled with DiI (1,1′‐dioctadecyl‐3,3,3′,3′‐tetramethyl indocarbocyanine, Invitrogen) to confirm their exact position after histological assessment (Nissl staining) post‐mortem. In PL, the most medial shank was confirmed to lay into layer II/III, whereas the most lateral shank was located in layer V/VI. In hippocampal CA1 area, the LFP reversal over stratum pyramidale was used for the selection of the channel with sharp waves of minimum amplitude and consequently, lowest contribution to the spectral content of the signal. Recordings that did not fulfil these criteria were not considered for analysis. One silver wire was inserted into cerebellum to serve as ground and reference electrode. A recovery period of 30 min following the insertion of electrodes before acquisition of data was provided. During surgery and recording, the body of the animal was kept at a constant temperature of 37°C using a heating blanket. Extracellular signals were band‐pass filtered (0.1 Hz to 8 kHz) and digitized (32 kHz) with a multi‐channel extracellular amplifier (Digital Lynx SX; Neuralynx) and the Cheetah acquisition software (Neuralynx). After recording, mice were anesthetized with 10% ketamine (WDT)/2% xylazine (WDT) in 0.9% NaCl solution (10 μg/g body weight, i.p.) and transcardially perfused with Histofix (Carl Roth) containing 4% paraformaldehyde. Brains were postfixed with Histofix for 24 hr and sectioned coronally at 100 μm. Wide‐field fluorescence images were acquired to reconstruct the recording electrode position in Nissl‐stained sections.

### 2.3 Data analysis

Data were imported and analysed offline using custom‐written tools in MATLAB software version 7.7 (MathWorks). The data were processed as following: (a) band‐pass filtered (500–5,000 Hz) to detect MUA as negative deflections exceeded five times the standard deviation of the filtered signals and (b) downsampled to 3,200 Hz before band‐pass filtering (3–100 Hz) to analyse the LFP. All filtering procedures were performed in a phase‐preserving manner. Frequency bands corresponded to the observed power peaks and ranged from 3–8 Hz (theta) to 12–30 Hz (beta).

#### 2.3.1 Detection of neonatal oscillatory activity

Discontinuous oscillatory events were detected using a previously developed unsupervised algorithm (Cichon, Denker, Grun, & Hanganu‐Opatz, 2014). Briefly, deflections of the root‐mean‐square of band‐pass (3–100 Hz) filtered signals exceeding a variance‐depending threshold were assigned as network oscillations. The threshold was determined by a Gaussian fit to the values ranging from 0 to the global maximum of the root‐mean‐square histogram. All consecutive oscillations with inter‐event intervals < 200 ms were considered as a single event. Only oscillatory events > 1 s were considered for further analysis. Time–frequency plots were calculated by transforming the data using the Morlet continuous wavelet.

#### 2.3.2 Power spectral density

For power spectral density analysis, 1‐s‐long windows of network oscillations were concatenated and the same was done for 1‐s‐long windows without oscillatory activity. For all detected oscillatory events *P*(*f*) and for all epochs without oscillatory activity *P*_0_(*f*), the absolute power was separately calculated using Welch's method (*pwelch.m*) with non‐overlapping hamming windows of 1 s length. Finally, the normalized (relative) power spectra were then defined as *P*(*f*)/*P*_0_(*f*).

#### 2.3.3 Spectral coherence

Coherence was calculated using the imaginary coherency method (Nolte et al., 2004). Briefly, the imaginary coherence was calculated by taking the imaginary component of the cross‐spectral density between the two signals (*cpsd.m*) normalized by the power spectral density (*pwelch.m*) of each. The computation of the imaginary coherence *C* over frequency (*f*) for the power spectral density *P* of signal *X* and *Y* was performed according to the formula:

$$C_{\mathrm{XY}}\left(f\right)=\left|\text{Im}\left(\frac{P_{\mathrm{XY}}\left(f\right)}{\sqrt{P_{\mathrm{XX}}\left(f\right)P_{\mathrm{YY}}\left(f\right)}}\right)\right|$$

#### 2.3.4 Generalized partial directed coherence

To investigate the directionality of functional connectivity between PL and i/vHP, generalized partial directed coherence (GPDC) were used. GPDC is based on linear Granger causality measure in the frequency domain. The method infers the causal relationship between simultaneously observed time series based on the decomposition of partial coherence computed from multivariate autoregressive models. The LFP signal was divided into 1‐s‐long segments containing the oscillatory activity. After detrending (*detrend.m*) and denoising (*wdencmp.m*), GPDC was calculated using a previously described algorithm (Baccalá, Sameshima, & Takahashi, 2007).

### 2.4 Statistics

Statistical analyses were performed in MATLAB. Data were fit to a linear model with weight and condition as terms (GLM, *fitglm.m*). Nested data as the MUA firing rate were analysed with a generalized linear mixed model (GLME, *fitglme.m*) with age and condition as fixed effects and animal as random effect. Regression terms (weight and age) were chosen on Bayesian model criterion. Distributions for GLME were indicated as a Poisson distribution, so data were automatically log‐transformed for the model. Overall sample set effect was tested with ANOVA (*anova.m*) and post hoc testing via contrast matrices (*coefTest.m*). To account for the multiple testing (six comparison), *p* values were post hoc corrected with the Benjamin–Hochberg method. Statistical testing was conducted on absolute values. To facilitate the comparison between different groups and their controls, the relative changes to the respective controls were displayed.

## 3 RESULTS

We investigated the early network function and coupling of PL and i/vHP at neonatal age in six mouse models of disease. To mimic the genetic background of disease, (a) mice carrying a human‐like truncating lesion in the endogenous murine DISC1 ortholog (DISC1^+/−^; Kvajo et al., 2008) and (b) mice carrying a chromosomal engineered 1.3‐Mb knockout syntenic to the 1.5‐Mb human 22q11.2 microdeletion (Df(16)A^+/−^; Stark et al., 2008) were investigated as one‐hit genetic models (DISC1 and Df16). To mimic the immune challenge during pregnancy, mice with prenatal immune activation by the viral mimetic poly I:C (Shi, Fatemi, Sidwell, & Patterson, 2003) were used as one‐hit environmental model (MIA). DISC1^+/−^ or Df(16)A^+/−^ mice prenatally treated with poly I:C recapitulated both genetic and environmental risk factors and were considered as dual‐hit gene–environment models (DISC1 + MIA and Df16 + MIA). Finally, mouse pups receiving subanaesthetic levels of ketamine from P1 to P8 were used as pharmacological model of disease (KET; Behrens et al., 2007). All six models have been extensively characterized at adulthood and shown to reproduce positive, negative and cognitive symptomology of schizophrenia (Abazyan et al., 2010; Krystal et al., 1994; Mukai et al., 2015; Niwa et al., 2010; Sigurdsson et al., 2010). Moreover, they have disrupted neuronal ensembles, abnormal prefrontal–hippocampal communication and neuronal dysfunction (Hamm et al., 2017).

### 3.1 Neonatal one‐hit Df16 and dual‐hit DISC1 + MIA mice show abnormal network activity and neuronal firing in PL

**Figure 1:**
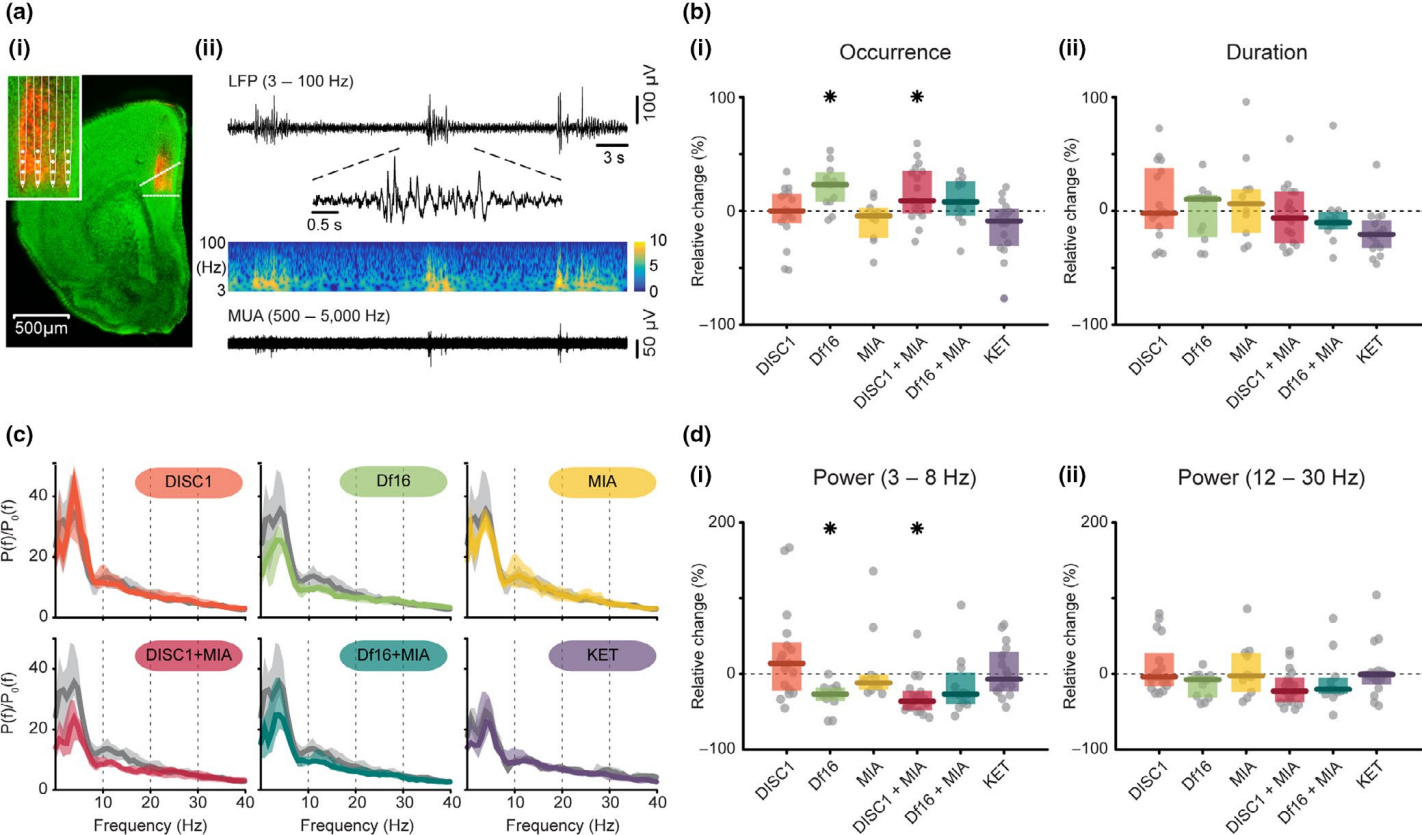
Patterns of oscillatory activity in the PL of neonatal mouse models of mental illness. (a) (i) Digital photomontage reconstructing the position of a 4‐shank DiI‐labelled recording probe (orange) in the PL of a Nissl‐stained 100 μm‐thick coronal section (green) from a P9 control mouse. Inset, the position of recording sites (white dots) over the prelimbic layers displayed at higher magnification. (ii) Extracellular recording of discontinuous oscillatory activity in PL from a P9 control mouse displayed after band‐pass (3–100 Hz) filtering accompanied by the colour‐coded wavelet spectra at identical timescale (middle) and the corresponding MUA after band‐pass (500–5,000 Hz) filtering (bottom). Inset, discontinuous oscillatory event displayed at higher magnification. (b) (i) Scatter plot displaying the relative occurrence of oscillatory events in PL of all models when normalized to controls (Df16 vs. control: *p *=* *0.01, DISC1 + MIA vs. control: *p *=* *0.01) (ii) Same for the duration of oscillatory events. (c) Averaged power spectra *P*(*f*) of discontinuous prelimbic oscillations normalized to the baseline power *P*_0_(*f*) of time windows lacking oscillatory activity displayed for all mouse models (colours) together with their control (light grey). (d) (i) Scatter plot displaying the relative prelimbic power within 3–8 Hz for all models when normalized to controls (Df16 vs. control: *p *=* *0.035, DISC1 + MIA vs. control: *p *=* *0.022) (ii) Same as (i) in the beta (12–30 Hz) frequency band. Thick lines represent the median and shaded areas represent the 25° and 75° percentiles. Single data points are represented as circles, the coloured bars represent the median and the coloured boxes the 25th and 75th percentiles, *p < 0.05

To test whether network dysfunction emerges already at neonatal age in all investigated models, we focused on the earliest developmental stage at which PL and CA1 area of i/vHP functionally interact. We performed extracellular recordings of local field potential (LFP) and multiple‐unit activity (MUA) from both areas in lightly anesthetized P8–10 controls (*n* = 23), saline controls (*n* = 13), DISC1 (*n* = 17), Df16 (*n* = 11), MIA (*n* = 10), DISC1 + MIA (*n* = 17), Df16 + MIA (*n* = 11) and KET (*n* = 16). In PL, the four shanks of recording electrodes were confirmed to be located across layer II/III and V/VI (Figure 1a(i)). Our previous investigations revealed that, despite reduction in the number of oscillatory events, their properties (power, frequencies distribution) and the neuronal firing are similar in urethane‐ and isoflurane‐anesthetized and asleep non‐anesthetized rodents of neonatal age (Bitzenhofer, Sieben, Siebert, Spehr, & Hanganu‐Opatz, 2015; Chini et al., 2019). Discontinuous (i.e., periods of network activity alternate with periods of “silence”) oscillatory discharges with frequency components peaking in theta (3–8 Hz) and beta‐low gamma frequency range (12–30 Hz) have been detected in all investigated mice (Figure 1a(ii)). Oscillatory activity with high frequency (>30 Hz) emerges later during development, hence is not detectable at P8–10. However, the oscillatory properties differed between groups (occurrence: *F*_7*,*111_ = 4.89, *p *<* *0.001; duration: *F*_7*,*111_ = 3.77, *p *<* *0.001; Figure 1b(i) and (ii), Table 1). In line with our previous findings (Hartung et al., 2016), the prelimbic activity of DISC1 + MIA mice appeared highly fragmented and correspondingly, the occurrence of oscillatory events was higher (*p *=* *0.01) when compared with controls. In contrast, the oscillatory activity in PL of one‐hit DISC1 and MIA mice was not affected. The fragmented structure of discharges was present also in one‐hit Df16 mice (*p *=* *0.01), yet the dysfunction did not substantially augment when MIA co‐occurred (Figure 1b(i) and (ii), Table 1). The relative power of oscillatory events normalized to the periods lacking coordinated activity was significantly decreased over theta band (3–8 Hz, *F*_7*,*111_ = 4.14, *p *<* *0.001) in DISC1 + MIA (*p *=* *0.022) and one‐hit Df16 (*p *=* *0.035) mice, yet not in the other groups, when compared to controls (Figure 1c and d(i), Table 1). In contrast, no significant differences were detected for faster frequencies (Figure 1d(ii)).

**Table 1:** Properties of discontinuous patterns of network activity in the PL of neonatal control and disease model mice

	Group effect		Control	Saline	DISC1	Df16	MIA	DISC1 + MIA	Df16 + MIA	KET
Occurrence (events/min)	ANOVA, *F*_7*,*111_ = 4.89; *p *<* *0.001***		7.07 ± 1.58	8.83 ± 1.53	7.07 ± 1.68	8.67 ± 1.84; ***p = 0.01*** *	6.77 ± 1.87	7.7 ± 2.59; ***p = 0.01*** *	7.63 ± 2.13	8.05 ± 2.83
Duration (s)	ANOVA, *F*_7,111_ = 3.77; *p *<* *0.001***		3.55 ± 1.62	4.06 ± 1.35	3.48 ± 1.66	3.66 ± 1.34	3.77 ± 1.36	3.33 ± 1.61	3.19 ± 0.54	3.22 ± 0.97
Rel. power (3–8 Hz)	ANOVA, *F*_7,111_ = 4.14; *p *<* *0.001***		25.43 ± 7.66	17.96 ± 5.58	28.88 ± 16.05	18.61 ± 4.27; ***p = *** **0.022** *	22.38 ± 4.91	16.17 ± 6.39; ***p *** **=** *** *** **0.035** *	18.58 ± 10.47	16.74 ± 9.32
Rel. power (12–30 Hz)			7.93 ± 3.45	6.87 ± 1.71	7.65 ± 3.4	7.35 ± 2.22	7.73 ± 4.1	6.15 ± 2.47	6.25 ± 1.66	6.87 ± 1.26
MUA firing rate (spikes/s)	ANOVA, *F*_7,494_ = 3.46; *p *=* *0.001**	LII/III	0.15 ± 0.48	0.19 ± 0.38	0.21 ± 0.35	0.4 ± 1.01	0.08 ± 0.56	0.06 ± 0.2; ***p = 0.008*** **	0.14 ± 0.33	0.1 ± 0.22
		LV/VI	0.22 ± 0.35	0.69 ± 0.6	0.26 ± 0.27	0.46 ± 0.41	0.2 ± 0.72	0.29 ± 0.37	0.17 ± 0.38	0.17 ± 0.44

Note

Data are shown as median ± interquartile range. Corrected *p* values (Benjamin‐Hochberg) for comparisons between controls and disease groups are listed. Italics indicate statistics of ANOVA for GLM/GLME; Italics and bolds indicate statistics of coefficient testing for GLM/GLME. *p < 0.05, **p < 0.01, ***p < 0.001.

The abnormal temporal organization of coordinated activity in the PL of dual‐hit DISC1 + MIA and one‐hit Df16 mice led us to hypothesize that the local prelimbic circuitry was perturbed in the two groups of mice. We calculated the firing rates in layer II/III and layer V/VI of the two models and compared them with the values from controls. Prelimbic neurons mostly fire during oscillatory events (Figure 1a). Overall, the firing in layer II/III but not V/VI of DISC1 + MIA mice decreased when compared to controls (*F*_7*,*494_ = 3.463, *p *=* *0.001, *p *=* *0.008; Figure 3a, b and Table 1). However, no significant changes were detected for the other groups.

Thus, combination of genetic and environmental risk as well as pronounced genetic abnormalities disrupt the prefrontal activity already at neonatal age, whereas early glutamatergic manipulation or single mild risk factors seem to not be sufficient for affecting neuronal ensembles in developing PL.

### 3.2 Neonatal one‐hit Df16 and dual‐hit mouse models show abnormal network activity in i/vHP

**Figure 2:**
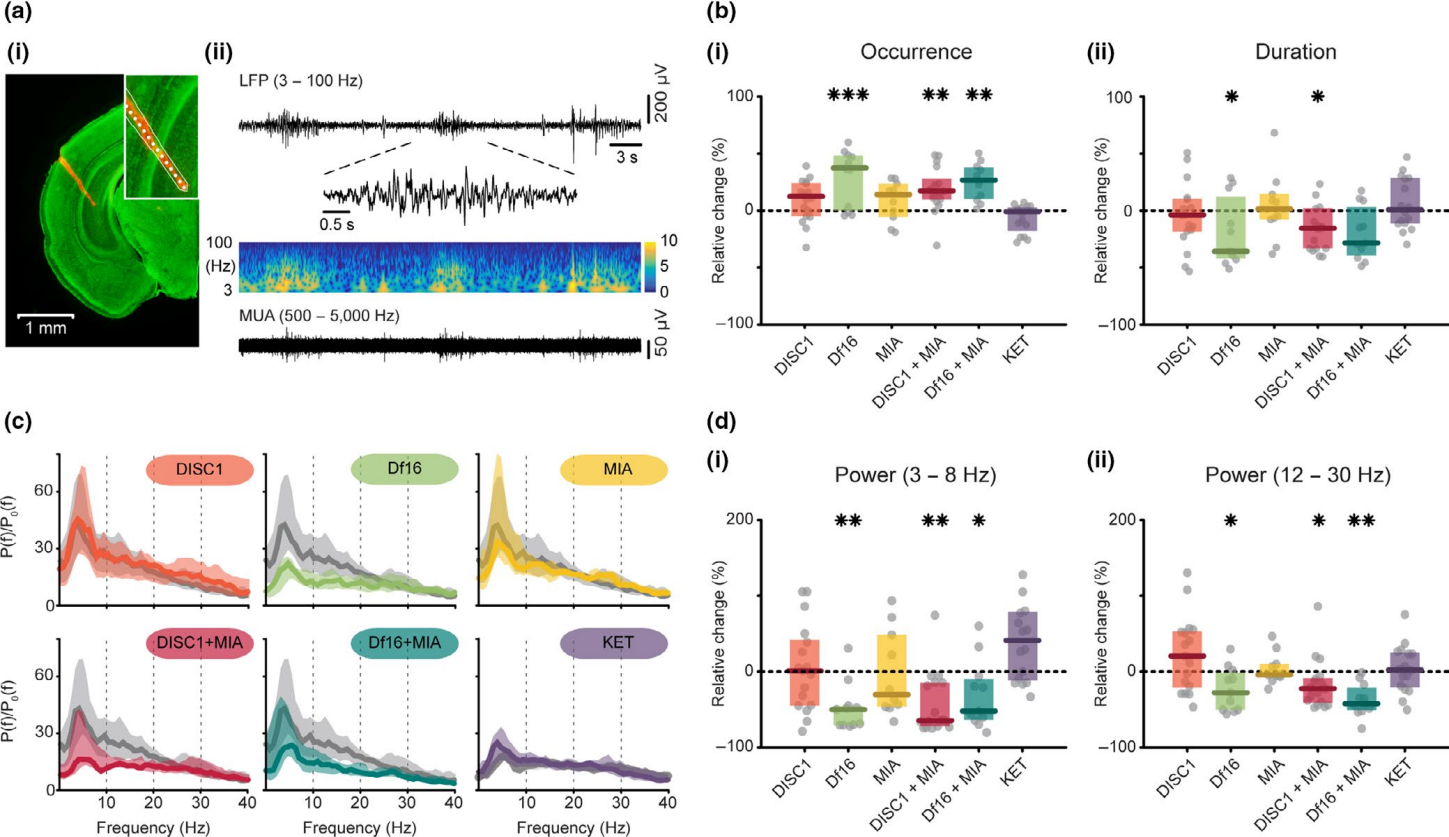
Patterns of oscillatory activity in the i/vHP of neonatal mouse models of mental illness. (a) (i) Digital photomontage reconstructing the position of a 1‐shank DiI‐labelled recording probe (orange) in the HP of a Nissl‐stained 100 μm‐thick coronal section (green) from a P9 control mouse. Inset, the position of recording sites (white dots) over the Str. pyramidale displayed at higher magnification. (ii) Extracellular recording of discontinuous oscillatory activity in HP from a P9 control mouse displayed after band‐pass (3–100 Hz) filtering accompanied by the colour‐coded wavelet spectra at identical timescale (middle) and the corresponding MUA after band‐pass (500–5,000 Hz) filtering (bottom). Inset, discontinuous oscillatory event displayed at higher magnification. (b) (i) Scatter plot displaying the relative occurrence of oscillatory events in hippocampal CA1 area of all models when normalized to controls (Df16 vs. control: *p *=* *0.001, DISC1 + MIA vs. control: *p *=* *0.002, Df16 + MIA vs. control: *p *=* *0.008). (ii) Same for the duration of oscillatory events (Df16 vs. control: *p *=* *0.018, DISC1 + MIA vs. control: *p *=* *0.018). (c) Averaged power spectra *P*(*f*) of discontinuous hippocampal oscillations normalized to the baseline power *P*_0_(*f*) of time windows lacking oscillatory activity displayed for all mouse models (colours) together with their control (light grey). (d) (i) Scatter plot displaying the relative hippocampal power within 3–8 Hz for all models when normalized to controls (Df16 vs. control: *p *=* *0.005, DISC1 + MIA vs. control: *p *=* *0.004, Df16 + MIA vs. control: *p *=* *0.043). (ii) Same as (i) in the beta (12–30 Hz) frequency band (Df16 vs. control: *p *=* *0.018, DISC1 + MIA vs. control: *p *=* *0.03, Df16 + MIA vs. control: *p *=* *0.002). Thick lines represent the median, and shaded areas represent the 25° and 75° percentiles. Single data points are represented as circles, the coloured bars represent the median and the coloured boxes the 25th and 75th percentiles, *p < 0.05, **p < 0.01, ***p < 0.001

Previous investigation identified discontinuous oscillatory activity in the CA1 area of i/vHP as drive of prefrontal entrainment at neonatal age (Ahlbeck, Song, Chini, Bitzenhofer, & Hanganu‐Opatz, 2018; Brockmann et al., 2011). Therefore, it is likely that the abnormal activity detected in the PL of one‐hit Df16 and dual‐hit DISC1 + MIA mice results not only from disrupted local coupling within prelimbic circuits, as shown by firing deficits, but also from abnormal communication with HP. This might result from either abnormal activity patterns in the neonatal HP of mouse models or impaired hippocampal drive to PL. To test these hypotheses, we monitored the discontinuous patterns of network activity in hippocampal CA1 area of all six models and compared them with those from controls. Hippocampal spindle‐shaped oscillations with main frequency in theta band and interspaced with faster beta/low‐gamma band discharges (Figure 2a) were analysed in their occurrence, duration and relative power.

**Figure 3:**
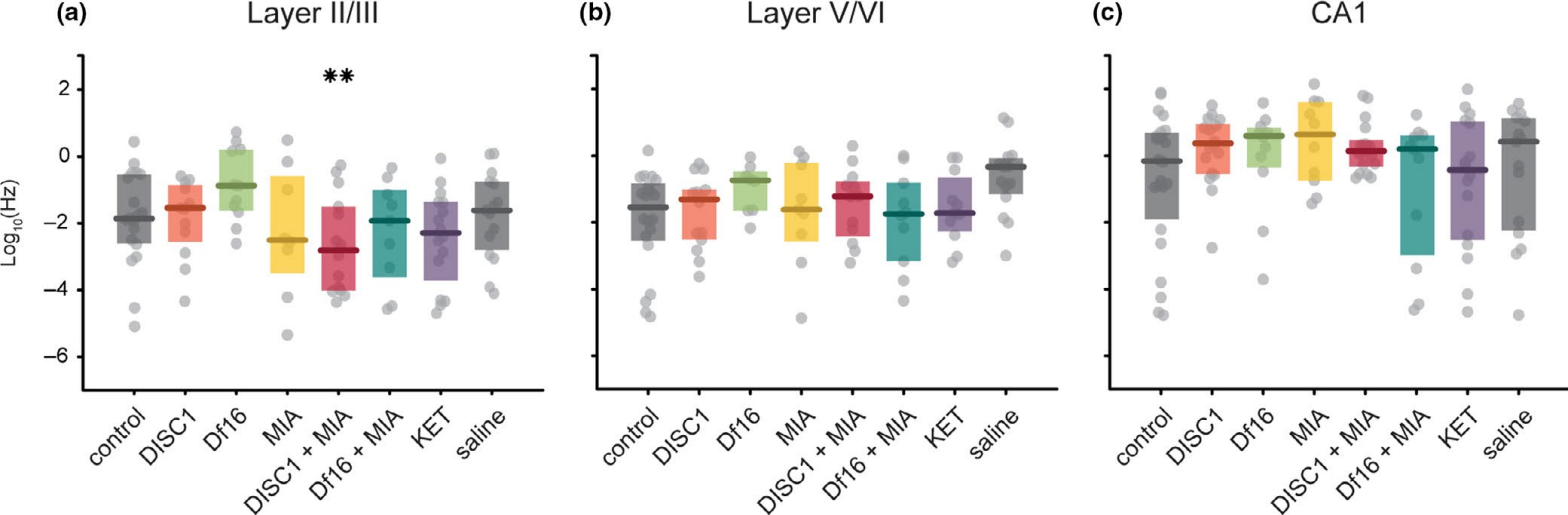
Firing rates of PL and i/vHP in mouse models of mental illness. (a) Scatter plot displaying the median firing rate in superficial layers of PL from all models (colours) and their controls (dark grey; DISC1 + MIA vs. control: *p *=* *0.008). (b) The same as (a) for deep layers of PL. (c) The same as (a) for CA1 area of i/vHP. Single data points are represented as circles, the coloured bars represent the median and the coloured boxes the 25th and 75th percentiles, **p < 0.01

Similar to PL, the most prominent fragmentation of hippocampal activity (i.e., higher occurrence and shorter duration) was detected for one‐hit Df16 and dual‐hit DISC1‐MIA mice (occurrence: *F*_7*,*111_ = 5.32, *p *<* *0.001; duration: *F*_7*,*111_ = 2.91, *p *=* *0.006; Figure 2b(i) and (ii), Table 2). Oscillations were significantly shorter in the DISC1 + MIA (*p *=* *0.018) and the one‐hit Df16 mice (*p *=* *0.018), and the oscillatory periods were more frequent (DISC1 + MIA: *p *=* *0.002; one‐hit Df16: *p *=* *0.001; Df16 + MIA: *p *=* *0.008). Their relative power was also significantly different when compared to controls (theta: *F*_7*,*111_ = 5.54, *p *<* *0.001; beta: *F*_7*,*111_ = 4.98, *p *<* *0.001; Figure 2c, d(i) and (ii) and Table 2). The DISC1 + MIA model showed a strong power reduction in theta (*p *=* *0.004, Figure 2d(i)) and beta band (*p *=* *0.03, Figure 2d(ii)). The power of the one‐hit Df16 and the Df16 + MIA was decreased in the theta (Df16: *p *=* *0.005; Df16 + MIA: *p *=* *0.043, Figure 2d(i)) and beta frequency range too (Df16: *p *=* *0.018; Df16 + MIA: *p *=* *0.002, Figure 2d(ii)). No significant changes in the occurrence, duration or power were detected in the other models when compared to controls. In all investigated mice, the hippocampal firing rate was comparable to that of controls (*p *=* *0.11, Figure 3c, Table 2).

**Table 2:** Properties of discontinuous patterns of network activity in i/vHP of neonatal control and disease model mice

	Group effect	Control	Saline	DISC1	Df16	MIA	DISC1 + MIA	Df16 + MIA	KET
Occurrence (events/min)	ANOVA, *F*_7,111_ = 5.32; *p *<* *0.001***	7.63 ± 2.5	10.3 ± 0.9	8.4 ± .1.84	10.13 ± 3.13; ***p = 0.001*** **	8.7 ± 1.8	9 ± 1.62; ***p = 0.002*** **	9.4 ± 2.03; ***p = 0.008*** **	9.38 ± 2
Duration (s)	ANOVA, *F*_7,111_ = 2.91; *p *=* *0.006**	4.49 ± 1.73	3.24 ± 1.02	4.09 ± 1.16	2.57 ± 2.13; ***p = 0.018*** *	4.42 ± 1.35	3.86 ± 1.4; ***p = 0.018*** *	3.91 ± 1.39	3.87 ± 1.03
Rel. power (3–8 Hz)	ANOVA, *F*_7,111_ = 5.54; *p *<* *0.001***	40.06 ± 25.97	15.06 ± 6.22	40.53 ± 34.53	19.99 ± 9.91; ***p = 0.005*** **	27.95 ± 37.95	14.18 ± 23.21; ***p = 0.004*** **	19.18 ± 21.47; ***p = 0.043*** *	21.28 ± 13.62
Rel. power (12–30 Hz)	ANOVA, *F*_7,111_ = 3.14; *p *<* *0.001***	16.5 ± 8.72	12.89 ± 3.89	19.86 ± 12.21	11.89 ± 8.1; ***p = 0.018*** *	15.8 ± 2.68	12.79 ± 5.29; ***p = 0.03*** *	9.54 ± 4.9; ***p = 0.002*** **	13.2 ± 5.91
MUA firing rate (spikes/s)		0.81 ± 1.77	1.46 ± 2.84	1.41 ± 1.98	1.7 ± 1.6	1.89 ± 4.45	1.1 ± 0.91	1.17 ± 1.72	0.63 ± 2.62

Note

Data are shown as median ± interquartile range. Corrected *p* values (Benjamin‐Hochberg) for comparisons between controls and disease groups are listed. Italics indicate statistics of ANOVA for GLM/GLME; Italics and bolds indicate statistics of coefficient testing for GLM/GLME. *p < 0.05, **p < 0.01, ***p < 0.001.

These findings indicate that the network activity in hippocampal CA1 area is compromised in neonatal one‐hit Df16 as well as dual‐hit mice. The absence of firing deficits in these mice confirms the minor contribution of CA1 neurons to the generation of discontinuous network oscillations (Janiesch, Kruger, Poschel, & Hanganu‐Opatz, 2011).

### 3.3 Neonatal one‐hit Df16 and dual‐hit mouse models show weaker long‐range coupling within prelimbic–hippocampal circuits

**Figure 4:**
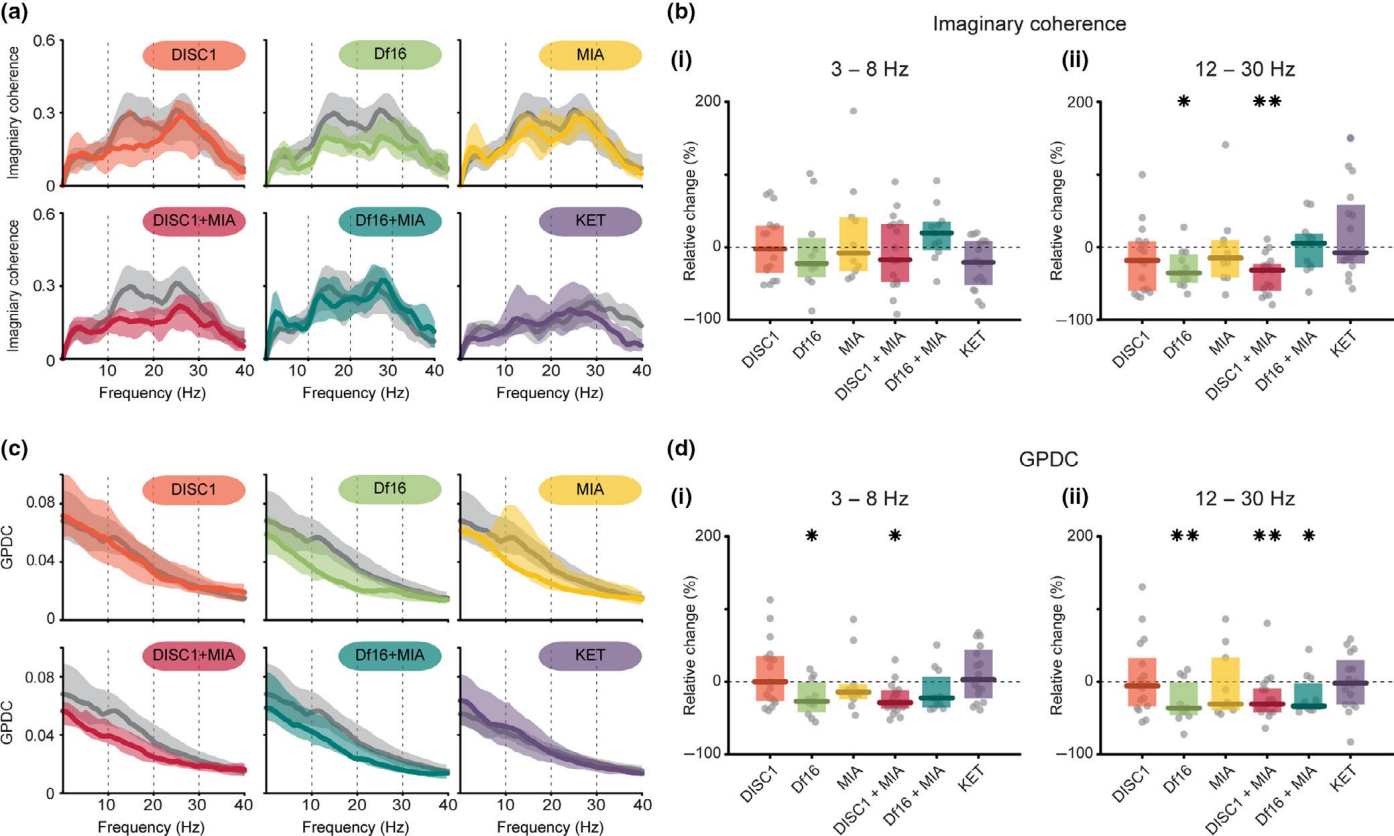
Coupling between PL and i/vHP in mouse models of mental illness. (a) Averaged coherence spectra *C*(*f*) for simultaneous oscillatory periods between PL and i/vHP displayed for all mouse models (colours) together with their control (light grey). (b) (i) Scatter plot displaying the relative coherence within 3–8 Hz for all models when normalized to controls. (ii) Same as (i) in the beta (12–30 Hz) frequency band (Df16 vs. control: *p* = 0.038, DISC1 + MIA vs. control: *p* = 0.001). (c) Averaged GPDC spectra *G*(*f*) from i/vHP to PL displayed for all mouse models (colours) together with their control (light grey). (d) (i) Scatter plot displaying the relative GPDC within 3–8 Hz for all models when normalized to controls (Df16 vs. control: *p* = 0.044, DISC1 + MIA vs. control: *p* = 0.01). (ii) Same as (i) for beta (12–30 Hz) frequency band (Df16 vs. control: *p* = 0.006, DISC1 + MIA vs. control: *p* = 0.006, Df16 + MIA vs. control: *p* = 0.017). Thick lines represent the median, and shaded areas represent the 25° and 75° percentiles. Single data points are represented as circles, the coloured bars represent the median and the coloured boxes the 25th and 75th percentiles, *p < 0.05, **p < 0.01

To test the hypothesis that abnormal communication between PL and HP is present in one‐hit Df16 and dual‐hit mice, we monitored the synchrony and information transfer between the two areas by calculating the coherence (Nolte et al., 2004) and generalized partial directed coherence (GPDC; Baccalá et al., 2007), respectively. We considered the imaginary part of coherence, which has the advantage over normal magnitude squared coherence of cancelling out any results due to volume conduction. Coherence was significantly altered in the beta range (*F*_7*,*111_ = 4.86, *p *<* *0.001), yet remained unchanged for 3–8 Hz (Figure 4b(ii), Table 3). The prelimbic–hippocampal synchrony was significantly reduced in one‐hit Df16 model (*p *=* *0.038) and dual‐hit DISC1 + MIA (*p *=* *0.001).

**Table 3:** Imaginary coherence and GPDC within prefrontal–hippocampal networks of neonatal control and disease model mice

	Group effect	Control	Saline	DISC1	Df16	MIA	DISC1 + MIA	Df16 + MIA	KET
Im. coherence (3–8 Hz)		0.13 ± 0.08	0.12 ± 0.02	0.12 ± 0.08	0.1 ± 0.07	0.12 ± 0.09	0.1 ± 0.1	0.15 ± 0.05	0.1 ± 0.08
Im. coherence (12–30 Hz)	ANOVA, *F*_7,111_ = 4.86; *p *<* *0.001***	0.26 ± 0.15	0.17 ± 0.1	0.21 ± 0.18	0.17 ± 0.1; ***p = 0.038*** *	0.22 ± 0.13	0.18 ± 0.09; ***p = 0.001*** **	0.28 ± 0.12	0.15 ± 0.13
GPDC (3–8 Hz)	ANOVA, *F*_7,111_ = 4.34; *p *<* *0.001***	0.063 ± 0.03	0.049 ± 0.011	0.063 ± 0.038	0.047 ± 0.025; ***p = 0.044*** *	0.054 ± 0.013	0.045 ± 0.016; ***p = 0.01*** *	0.049 ± 0.027	0.05 ± 0.033
GPDC (12–30 Hz)	ANOVA, *F*_7,111_ = 3.14; *p *=* *0.003**	0.037 ± 0.015	0.029 ± 0.009	0.034 ± 0.024	0.023 ± 0.016; ***p = 0.006*** **	0.025 ± 0.026	0.025 ± 0.012; ***p = 0.017*** *	0.024 ± 0.013; ***p = 0.006*** **	0.029 ± 0.018

Note

Data are shown as median ± interquartile range. Corrected *p* values (Benjamin‐Hochberg) for comparisons between controls and disease groups are listed. Italics indicate statistics of ANOVA for GLM/GLME; Italics and bolds indicate statistics of coefficient testing for GLM/GLME. *p < 0.05, **p < 0.01, ***p< *0.001*.

Next, we estimated the communication within prelimbic–hippocampal networks by GPDC that is considered to be a more precise measure of coupling, since it takes the time dimension of information transfer into account (Baccalá et al., 2007). We analysed the drive from i/vHP to PL in all investigated groups (Figure 4c, d). GPDC was over the sample set significantly altered in the theta and beta range (theta: *F*_7*,*111_ = 4.34, *p *<* *0.001; beta: *F*_7*,*111_ = 3.14, *p *=* *0.003). Significant decrease in hippocampal drive was detected in one‐hit Df16 mice (theta: *p *=* *0.044; beta: *p *=* *0.006) as well as in dual‐hit DISC1 + MIA (theta: *p *=* *0.01, beta: *p* = 0.006) and Df16 + MIA (beta: *p *=* *0.017; Table 3). The synchrony and directed interactions in one‐hit DISC1, MIA as well as in KET mice were similar to controls.

These results demonstrate that developmental deficits of local circuitry in PL and HP in one‐hit Df16 and dual‐hit mice accompany weaker coupling within neonatal prelimbic–hippocampal networks. In contrast, early dysfunction of these circuits was not present in mice reproducing only one risk factor of disease (e.g., DISC1 or MIA) or in pharmacological model (i.e., KET).

## 4 DISCUSSION

A wealth of rodent models has been proposed to mimic aspects of human mental disorders. Their large number and diversity lead to the question whether common mechanisms cause in the end the characteristic symptoms of disease. For neurodevelopmental disorders, such as schizophrenia, diverse risk factors might similarly affect the brain by acting on a common pathway that disturbs network maturation, resulting in detectable cognitive deficits at juvenile‐young adult stage. To examine this hypothesis, we investigated the neurodevelopmental abnormalities in six distinct animal models of disease whose adult phenotype has been previously characterized in detail. We focused on the core circuit underlying memory and executive abilities, the prelimbic–hippocampal network, and showed that shortly after birth, the early patterns of coordinated activity in PL and HP appear disorganized with lower power in mice modelling 22q11.2 microdeletions, a genetic variant highly penetrant for schizophrenia, and mice combining genetic dysfunction with an environmental stressor (DISC1 + MIA, Df16 + MIA). Additionally, these mice showed weaker synchrony and directed coupling between PL and HP, especially in beta frequency band. In contrast, no significant prelimbic–hippocampal deficits were observed in one‐hit (DISC1, MIA) or KET mice.

Since at adulthood all investigated mice have been used as models of disease and mimic, at least in part, the dysfunction and cognitive impairment of mental illness, it is likely that the disease‐related miswiring of neuronal circuits emerges at different developmental time points. For example, the disruption of limbic circuits centred on the prefrontal–hippocampal networks and the impairment of memory and executive abilities have been previously reported in DISC1 haploinsufficiency, transgenic and point mutations models (Crabtree et al., 2017; Dawson et al., 2015; Koike, Arguello, Kvajo, Karayiorgou, & Gogos, 2006). Specifically, the dysfunction of local and long‐range circuits centred on PFC correlate with decreased working memory, prepulse inhibition, sociability, and spatial recognition memory as well as increased depression‐like behaviour (Clapcote et al., 2007; Niwa et al., 2010; Sauer, Strüber, & Bartos, 2015). However, in line with our past (Hartung et al., 2016) and present results, the genetic abnormality is not sufficient for disrupting the initial entrainment of neonatal prefrontal–hippocampal circuits in oscillatory rhythms.

A second stressor is necessary to induce early dysfunction. MIA mimicked by treatment with polyI:C at distinct gestational stages has been identified as environmental factor linked to schizophrenia. Adult MIA rodents show circuit abnormalities (interneuronal deficits and diminished gamma oscillations in PFC) and decrease long‐range coupling over a broad frequency spectrum within prefrontal–hippocampal circuits (Dickerson, Wolff, & Bilkey, 2010). Consequently, they show poorer PFC‐dependent working memory, prepulse inhibition, cognitive flexibility and attentional set‐shifting abilities (Canetta et al., 2016; Meyer & Feldon, 2012). While neonatal MIA mice were almost indistinguishable from controls, in combination with the abnormal DISC1 background, they show profound impairment of early prelimbic–hippocampal communication. Both brain areas have disorganized activity patterns that occur more frequently but have reduced power in theta and beta‐low gamma frequency range. These alterations of network activity might reflect a global perturbation of brain activity due to the combined action of genetic and environmental factors on cortical migration and differentiation. Disc1 mutation may induce perturbation of immune‐relevant signalling pathways early in life (Beurel, Yeh, Michalek, Harrington, & Jope, 2011). The disorganized activation of HP and the weaker drive to PL is accompanied by a reduction in axonal projections between the two areas (Song and Hanganu‐Opatz, unpublished observations). These structural and functional deficits may perturb the prelimbic activity and initial wiring of local circuits. Structural changes in PL, such as decrease in spine density or lower number of axonal terminals, might lead to decreased firing of pyramidal neurons in PL (Xu et al., 2019), but also to loss of theta‐timed precision of firing, as reflected by the weaker strength of theta‐spike coupling (Hartung et al., 2016). These results are in line with previous data reported for adult dual‐hit DISC1‐MIA mice that show decreased spine density on hippocampal neurons as well as decreased sociability and augmented anxiety and depressive‐like behaviour (Abazyan et al., 2010).

Surprisingly, similar potentiating effects of combined genetic environmental factors were not observed for the dual‐hit Df16 + MIA mice. This might be due to the fact that the genetic abnormality mimicking the 22q11.2 microdeletions has already a maximal impact on the early circuits, in contrast to the rather mild phenotype of Disc1 mutation. Conserved on mouse chromosome 16, these microdeletions affect several key genes, such as Dgcr8, Comt and Prodh that even alone affect dendritic development and dopamine metabolism (Arguello, Markx, Gogos, & Karayiorgou, 2010; Paterlini et al., 2005). The present data demonstrate that the prefrontal and hippocampal activity of one‐hit Df16 mice is altered in a similar way as that of dual‐hit DISC1 + MIA mice. The higher occurrence of shorter low power oscillations in both areas was accompanied by weaker synchrony and diminished drive from HP to PL at neonatal age. This early circuit miswiring most likely result from abnormal physiological properties of cortical neurons that have identified in vitro already at embryonic stage (Sun, Williams, Xu, & Gogos, 2018). Adult Df(16)A^+/−^ mice have working memory deficits and impaired conditioned fear as result of reduced coupling within prefrontal–hippocampal networks (Sigurdsson et al., 2010; Stark et al., 2008). Co‐occurrence of MIA does not augment but even reduce the deficits observed in the one‐hit Df16 mice. Whether poly I:C partially rescues the morphological and functional abnormalities of microdeletions remains to be elucidated in future studies. One possible mechanism may involve micro RNAi that are upregulated in the dam by MIA (Hollins & Cairns, 2016) and may cross the placenta barrier (Chang et al., 2017), compensating for microdeletions (Stark et al., 2008).

Neonatal KET mice showed no prelimbic–hippocampal deficits when compared with their saline control. Adult mice that received chronic ketamine administration have been reported to have altered neuronal ensembles as result of pyramidal and interneuronal dysfunction (Behrens et al., 2007; Hamm et al., 2017), while acute administration leads to spectral alterations of the prefrontal–hippocampal circuitry (Moran et al., 2015). Consequently, these mice have poorer memory performance, decreased mismatch negativity and cognitive flexibility as well as altered social behaviour (Amitai, Semenova, & Markou, 2007; Featherstone et al., 2012; Hauser, Isbrandt, & Roeper, 2017; Koh, Shao, Sherwood, & Smith, 2016). The similar outcome of NMDA receptor blockade or saline treatment during neonatal period may indicate that the disease‐related glutamatergic dysfunction emerges later and perturbs the neuronal circuits after the establishment of their first interactions. In line with this hypothesis, ketamine administration during the 2nd post‐natal week leads to reduced PV expression in PFC and attentional, memory and social deficits (Jeevakumar et al., 2015).

Thus, the present results show that common deficits reported for different mouse models of mental disorders may share common pathways of impairment. The prefrontal–hippocampal communication critically controlling the cognitive performance is a hub of disease‐induced impairment, yet sensory circuits and related abilities might be disrupted too (Hamm et al., 2017). However, depending on the severity of risk factors this hub is susceptible to impairment during a different time window in life. This temporal aspect needs to be considered when aiming to assemble the puzzle for understanding mental disorders.

## CONFLICT OF INTEREST

The authors declare no competing financial interests.

## AUTHOR CONTRIBUTIONS

I.L.H.‐O. designed the experiments; V.O. carried out the experiments; V.O., X.X. and M.C. analysed the data; I.L.H.‐O., X.X. and V.O. interpreted the data and wrote the paper. All authors discussed and commented on the manuscript.

## Data Availability

The authors declare that all data and code supporting the findings of this study are included in the manuscript or are available from the corresponding author on request.
